# Cervical Cancer and Fertility-Sparing Treatment

**DOI:** 10.3390/jcm10214825

**Published:** 2021-10-21

**Authors:** François Zaccarini, Claire Sanson, Amandine Maulard, Stéphanie Schérier, Alexandra Leary, Patricia Pautier, Cyrus Chargari, Catherine Genestie, Sébastien Gouy, Philippe Morice

**Affiliations:** 1 Department of Gynecologic Surgery, Gustave Roussy, 94800 Villejuif, France; claire.sanson@gustaveroussy.fr (C.S.); amandine.maulard@gustaveroussy.fr (A.M.); stephanie.scherier@gustaveroussy.fr (S.S.); sebastien.gouy@gustaveroussy.fr (S.G.); philippe.morice@gustaveroussy.fr (P.M.); 2 Department of Medical Oncology, Gustave Roussy, 94800 Villejuif, France; alexandra.leary@gustaveroussy.fr (A.L.); patricia.pautier@gustaveroussy.fr (P.P.); 3 Department of Radiation Oncology, Gustave Roussy, 94800 Villejuif, France; cyrus.chargari@gustaveroussy.fr; 4 Department of Pathology, Gustave Roussy, 94800 Villejuif, France; catherine.genestie@gustaveroussy.fr

**Keywords:** early-stage cervical cancer, fertility-sparing treatment, conization, trachelectomy

## Abstract

Radical hysterectomy with pelvic node dissection is the standard treatment for early-stage cervical cancer. However, the latter can be diagnosed at a young age when patients have not yet achieved their pregnancy plans. Dargent first described the vaginal radical trachelectomy for patients with tumors <2 cm. It has since been described a population of low risk of recurrence: patients with tumors <2 cm, without deep stromal infiltration, without lymphovascular invasion (LVSI), and with negative lymph nodes. These patients can benefit from a less radical surgery such as conization or simple trachelectomy with the evaluation of the pelvic node status. Tumors larger than 2 cm have a higher risk of recurrence and their treatment is a challenge. There are currently two options for these patients: abdominal radical trachelectomy or neoadjuvant chemotherapy (NACT), followed by fertility-sparing surgery. All patients who wish to preserve their fertility must be referred to expert centers.

## 1. Introduction

Despite prevention screening campaigns for cervical cancer and the widespread of HPV-vaccination among several countries, this disease remains the fourth most common cancer in women worldwide [1]. International Federation of Gynecology and Obstetrics (FIGO) stage determines the most appropriate treatment, and radical hysterectomy is currently the standard therapy for patients at early stage (2018 FIGO IA2 to IB2 and FIGO IIA1) [2]. However, according to the SEER (Surveillance, Epidemiology and End Results) Program in the United States, 36% of patients with a cervical cancer are under 45 years old. Some of these women wish to preserve their fertility. Dargent described in 1986 the radical vaginal trachelectomy with bilateral pelvic lymphadenectomy for tumors <2 cm [3]. Since the survival rates of this procedure are comparable to radical hysterectomy for early-stage cervical cancers, it is one of the standard fertility-sparing treatment. For several years, we have been trying to find the best oncological treatment associated with less morbidity. Radical trachelectomy is known to increase miscarriage and preterm delivery. Other surgical procedures such as conization and simple trachelectomy have been used to minimize the obstetrical morbidity of radical trachelectomy. Conization or simple hysterectomy are the recommended treatments for stage IA1 (FIGO 2018) without LVSI (lympho-vascular space involvement). Different treatment options are available according to the NCCN and ESGO recommendations for FIGO 2018 stages IA1 with LVSI, IA2 and IB1. The aim of this study is to perform a summary of the different existing conservative treatments for early-stage cervical cancer from FIGO 2018 IA with LVSI to IB2 stages.

## 2. Dargent’s Procedure (Vaginal Radical Trachelectomy)

Plante et al. [4] and then Marchiole et al. [5] demonstrated the safety of Dargent’s procedure for patients with tumors <2 cm. After using a propensity score matching in a population of 15,150 patients from the National Cancer Database undergoing hysterectomy or trachelectomy for IA2-IB2 (FIGO 2018) cervical cancer, Cui et al. [6] showed that there was no association between radical trachelectomy and the risk of mortality. There was also no difference in the 5-year overall survival rates between trachelectomy and hysterectomy. In this study, almost 30% of the FIGO IB population were classified as “IB not otherwise specified”, and the use of neoadjuvant chemotherapy was not specified. Due to the database used, data regarding the free-disease survival were not available.

Patients with tumors >2 cm are not appropriate candidates for Dargent’s procedure, since the risk of recurrence is high. In a review published in 2016 [7], the recurrence rate among patients with tumors >2 cm was 17% compared with 4% among patients with stage IB tumors ≤2 cm.

The purpose of parametrectomy, for some selected patients who have a low risk of parametrial invasion, has been questioned for several years. Parametrectomy can cause urinary and digestive morbidities by denervation of the inferior hypogastric plexus [8]. The rate of parametrial involvement is less than 1% among patients with tumors ≤2 cm, without LVSI, with negative lymph nodes, with stromal invasion ≤10 mm [9]. The SHAPE trial [10] currently underway will answer the need of parametrectomy in low-risk patients undergoing hysterectomy. Furthermore, it is hypothesized that parametrectomy can increase the risk of miscarriage and preterm delivery. For all these reasons, less radical treatments have been used for several years for selected patients.

Dargent’s procedure requires training and experience. Due to the difficulty of this procedure and the advent of the laparoscopy, other approaches have been developed to perform radical trachelectomy.

## 3. Abdominal Trachelectomy, Laparoscopic Radical Trachelectomy and Vaginal Radical Trachelectomy

Since Dargent’s procedure, others’ approaches of radical tracheclectomy have been described, such as abdominal radical trachelectomy or minimally invasive radical trachelectomy (laparoscopic or robot-assisted). These approaches are thought to be more radical in terms of parametrial resection; they could be proposed to patients with tumors with unfavorable prognosis (tumor size > 2 cm, LVSI). Practices of some teams have changed since the LACC trial [11], even if this study did not focus on fertility-sparing treatments. 

Bentivegna et al. [7] reported 866 patients who had abdominal radical trachelectomy. Among those, 559 patients had a 2008 FIGO-stage IB1 tumors, of which at least 167 tumors were larger than 2 cm. Recurrence rate was 5% in all patients and 5% in patients with tumors measuring 2 to 4 cm. Laparoscopic radical trachelectomy was described in 238 patients, of which 6% had recurrent disease. However, 17% of the said recurrent disease were noticed in tumors larger than 2 cm.

A recent review reported 2566 patients undergoing vaginal radical trachelectomy (58.1%), abdominal radical trachelectomy (37.2%) and minimally invasive radical trachelectomy (4.7%) [12]. There was no difference in recurrence rate and 5 year-overall survival. However, the operative time was shorter, and there were lower rates of positive margins in case of vaginal approach.

The obstetrical outcomes of these different techniques are described in a specific chapter. It is difficult to draw some conclusions and to recommend a specific approach. However, it seems reasonable to advise to perform a vaginal radical trachelectomy. 

## 4. Conization or Simple Trachelectomy with Pelvic Lymph Node Dissection

Conization or simple hysterectomy are the recommended treatments for stage IA1 (FIGO 2019) without LVSI. Different treatment options are available according to the NCCN and ESGO recommendations for stages IA1 with LVSI, IA2 and IB1. Conization, simple trachelectomy or radical trachelectomy are accepted treatments in association with pelvic lymph node dissection or sentinel lymph node biopsy. Bentivegna et al. [7] reported the data from 159 articles about conservative treatment for cervical cancer. Two hundred and thirty patients among 13 series with stage IB1 tumors ≤2 cm lymph node negative who had simple trachelectomy or conization with pelvic lymph-node dissection were counted. Only six patients had a recurrence. In this review, they proposed a treatment algorithm for management of node-negative stage IB1 (FIGO 2009) cervical cancer in which patients with tumors <2 cm without LVSI could benefit from a conization or a simple trachelectomy, while patients with tumors <2 cm with LVSI should have a vaginal radical trachelectomy.

A study using the SEER database showed that there was no difference in disease-specific survivals between 807 patients treated with less radical surgery (conization, trachelectomy or hysterectomy) and 1764 patients treated with radical surgeries, after stratification on tumor size (≤2 cm or >2 cm) [13]. However, there is a major risk to use adjuvant therapies in patients with tumors >2 cm. Unfortunately, the impact of LVSI was not studied.

Bogani et al. [14] showed that there was no recurrence disease among patients undergoing conization and pelvic node dissection with negative lymph nodes. This study included 32 patients with FIGO (2018) IA2, IB1 and IB2 tumors with 48% of patients having LVSI. The median follow-up was 75 months. A study recently published by Plante et al. [15] reported 42 simple trachelectomies and eight conizations with laparoscopic sentinel lymph node mapping ± complete pelvic node dissection for patients with tumors <2 cm and wishing to preserve their fertility. The 5-year progression-free and overall survival were 97.9% and 97.6%, respectively. One patient had a recurrence and eventually died. In the 10-year single institution experience published by Tomao [16], there were 7 recurrences (13%) out of 54 patients undergoing conization and pelvic lymphadenectomy. LVSI was present in 22.2% of patients. Six of the recurrences were local. Among the seven recurrences, two patients had LVSI. In a serie of 40 patients [17] undergoing conization and laparoscopic pelvic lymphadenectomy for tumors <2 cm, only one patient had a recurrence during a median follow-up of 35 months. LVSI was present in 37.5% of cases. Other previous studies showed low rates of recurrence in case of conization [18,19,20]. 

A review and meta-analysis by Zhang et al. [21] showed that among 2854 patients undergoing conization for stage FIGO 2008 IA tumors and IB tumors, recurrence rates were 0.4 and 0.6%, respectively. Another recent review listed the outcomes of the different fertility-sparing surgeries (conization, vaginal radical trachelectomy, abdominal trachelectomy and mini-invasive trachelectomy) in 53 studies [22]. The recurrence rate for conization, vaginal radical trachelectomy, abdominal trachelectomy and mini-invasive trachelectomy was 4.2%, 4%, 3.9% and 4.2%. However, patients with FIGO 2009 IIA cervical cancer and with positive lymph nodes were listed, and it is unclear whether there were included in the statistical analysis.

The results of the Concerv trial [23] were recently published. One hundred women with a low-risk early-stage cervical cancer (tumor < 2 cm, no LVSI, depth of invasion <10 mm and cone biopsy with negative margins) had a conization (44%) or a simple hysterectomy (56%) with pelvic lymph node assessment. Three patients (3%) had a recurrence and the median follow-up was 36 months: two patients with IB1 disease and one with an IA2 tumor.

The impact of LVSI is unclear. Should the presence of LVSI in FIGO 2018 stage-IB1 tumors be an indication of radical trachelectomy as it was advised in Bentivegna’s review in 2016? A recent Japanese [24] study proposed different models to select patients for fertility-sparing trachelectomy; LVSI was not part of the final model that included the histology (squamous cell carcinoma, adenocarcinoma or adenosquamous carcinoma), tumor size ≤2 cm, nodal metastases and deep stromal invasion. The prospective GOG study [25] published in 1990 and other more recent studies showed that disease free-survival was worse among patients with LVSI [26,27], in contrast to a review published in 2004 [28]. However, most studies about cervical cancer are only mentioning the presence or absence of LVSI. It seems that a more complex evaluation of LVSI should be conducted. Indeed the localization and the quantification of LVSI could play an important role and should help discriminate patients with a higher risk of relapse. First, Herr et al. [29] described in 281 patients that satellite LVSI, defined as LVSI occurring at a minimal distance of 1 cm from the tumor, was associated with a decreased overall and disease-free survival, which was not observed in presence of conjoined LVSI alone (LVSI in close proximity to the tumor invasion front). Pol et al. [30] confirmed that satellite LVSI was an independent factor for recurrence and survival. Recently, Ronsini et al. [31] described a semi-quantitative evaluation of LVSI, divided as absent (no LVSI), focal (a single focus of LVSI), and diffuse (more than 1 LVSI around the tumor). Among 750 patients with 2018 FIGO stages-IA1 with LVSI to IIIC, patients with diffuse LVSI had a significantly higher risk of positive lymph nodes, and parametrial involvement than patients without LVSI. Patients with focal LVSI were comparable to patients without LVSI. Similarly, disease-free survival and overall survival were lower in presence of LVSI, but this difference was not found in presence of focal LVSI alone. For information, 146 patients had a FIGO stage-IB1 tumor, in which 39.1% had focal LVSI and 19.8% had diffuse LVSI. Another issue regarding LVSI is the way they are evaluated. Indeed, it has been recently proven that the diagnosis of LVSI suffers from a high inter and intra-rater variability [32]. This raises the question of the importance given to LVSI in the choices of both conservative and adjuvant treatments. The histological material should be reanalyzed by a trained pathologist before any treatment decision.

Negative surgical margins are obviously essential for conservative treatment. There is no clear definition of close or appropriate margins in the litterature. Positive margins are rarely found to be an independent factor of poor prognostic [33] in the literature but if it is associated with others’ high-risk factors [34].

There are more and more arguments in favor of less radical surgery for FIGO 2018 stage IA2 and IB1 tumors. Lymph node status must be known to validate a fertility-sparing treatment. Total pelvic lymph node dissection or sentinel lymph node biopsy should be performed at the same time of conization or simple trachelectomy. A vaginal radical trachelectomy should be discussed in the presence of risk factors such as deep stromal invasion or LVSI.

## 5. Neoadjuvant Chemotherapy (NACT) Followed by Vaginal Radical Trachelectomy or Abdominal Radical Trachelectomy for Tumors >2 cm

Patients with FIGO 2018 stage-IB2 cervical cancer who wish to preserve their fertility have a high risk of relapse. They must be referred to expert centers where they can be offered two approaches: NACT followed by vaginal radical trachelectomy with pelvic lymphadenectomy before chemotherapy, or abdominal radical trachelectomy with pelvic lymph node dissection.

Bentivegna et al. reported, in 2016 in a review of the literature [7], 52 cases of NACT followed by conservative surgery, and 209 cases of abdominal radical trachelectomy. The rate of recurrences was respectively 6% and 7%. However, they suspected that the recurrence rate in case of NACT was underestimated. Van Kol et al. published a review [35] comparing these two treatments; 338 patients were identified in the literature. After NACT followed by vaginal radical trachelectomy, the recurrence rate was 10% and the death rate was 2.9%. Among these patients, only 39% tried to conceive, of which 70% became pregnant, and 63% had a living birth. After abdominal radical trachelectomy, the recurrence rate was 6.9% and the death rate was 3.4%. Forty percent of these patients tried to conceive. Among them, 21% became pregnant with a life birth rate of 42%.

Another review published recently listed 249 patients with FIGO 2018 stage-IB2 cervical cancer and undergoing NACT followed by fertility-sparing surgery. The recurrence rate was 6.1%; two patients died (1.8%) of disease. There were 64 pregnancies, of which 49 (76.6%) living birth with six preterm births (9.4%). Burbano et al. [36] recently showed a recurrence rate of 12.8% and a 2.8% death rate in case of NACT for tumors larger than 2 cm. This review included 205 patients.

The ongoing CONTESSA trial that includes patients under 40 years old with FIGO 2018 IB2-cervical cancer receiving NACT before a conservative surgery will evaluate the rate of successful fertility-sparing surgery.

The best strategy for these patients remains unclear. Latest data seem to show recurrence rate around 10% sometimes more in case of NACT. Is a 10% risk of relapse acceptable, even if the obstetrical outcomes seem to be better? Lymph node status should be evaluated prior to NACT, using sentinel lymph node biopsy and pelvic lymphadenectomy, to exclude patients who have positive lymph nodes for fertility-sparing treatments. However, some authors recommend performing a single surgery with lymphadenectomy after NACT, arguing that it does not affect the recurrence rate [36]. However, abdominal radical trachelectomy seems to offer a better oncological control. Patients who wish to preserve their fertility should be addressed to expert centers, so that a change of strategy can be made quickly in case of early relapse.

## 6. Obstetrical Outcomes

The advantage of a less-radical surgery should increase the chance of pregnancy for patients treated for early-stage cervical cancer. Zhang’s review of the literature and meta-analyses [21], in addition to demonstrating low recurrence rates with conization, appears to show better oncological outcomes. The pregnancy, miscarriage and premature birth rates were 36.1%, 14.8% and 6.8% compared to 20.5%, 24% and 26.6% in case of trachelectomy.

In 2020, Nezhat et al. [37] reported 3044 patients who had fertility-sparing surgery, of which 40% (1218) tried to conceive for a result of 1047 pregnancies. There was a significant difference of pregnancy rate between vaginal radical trachelectomy (67.5%) and abdominal radical trachelectomy (41.9%). Conization and simple trachelectomy showed higher live-birth rate (86.4%), compared to abdominal radical trachelectomy (65.7%), vaginal radical trachelectomy (63.4%) and laparoscopic radical trachelectomy with or without robotic assistance (56.5%). Preterm delivery rate was 31.0% for all patients with 25.1% preterm delivery after conization or simple trachelectomy, and 34.6%, 30.5% and 31.4% after vaginal, abdominal and laparoscopic radical trachelectomies.

In the recent review published by Smith et al. [12], the pregnancy rate was 37.8%, 10.4% and 9.2% after vaginal, abdominal and laparoscopic radical trachelectomies with a live-birth rate of 75.7%, 75.6% and 57.1%, respectively. Preterm delivery happened in 33.9%, 39% and 57.1% of cases after vaginal, abdominal and laparoscopic radical trachelectomies.

Pregnancies after fertility-sparing treatments have higher risk of complication such as premature rupture of membranes and preterm delivery, which emphasizes the need to refer these patients to specialists in maternal-fetal medicine.

## 7. Follow-Up

The literature regarding the follow-up of patients after a fertility-sparing treatment is very poor. In a study including 43 patients with FIGO 2008 IA2 to IB2 cervical cancer who underwent a fertility-sparing surgery, colposcopy alone and in association with HPV positivity showed the best sensitivity to predict recurrence [38]. Cytology after fertility-sparing surgery can be a cause for useless concern. Their results can show abnormalities without any recurrence. Post-operative changes make the interpretation of Pap smear difficult. In a series of 44 patients, 223 cytology specimens were studied after radical trachelectomy [39]. An endometrial component was identified in 131 cases (59%) and 28 cytologies were abnormal. Only four lesions (three low-grade squamous intraepithelial lesions and one adenoquamous carcinoma) were diagnosed. HPV testing should be done at the first follow-up visit at 3 months, then at 6 months and 12 months.

MRI remains the best imaging method after fertility-sparing treatments and must be analyzed by an experienced radiologist, because of the postoperative imaging appearance of the remaining cervix. MRI monitoring can be performed routinely at 6 and 12 months, for example, or in the event of clinical suspicion.

## 8. Conclusions

Fertility preservation treatments for patients with early-stage cervical cancer do not put patients at higher risk of recurrence if they are well selected. Conization or simple trachelectomy with pelvic node dissection should be performed for patients with a low risk of recurrence. The presence of risk factors such as LVSI and deep stromal infiltration should lead to the realization of a vaginal radical trachelectomy. However, some work about a more precise characterization of LVSI should be engaged to select the patients better. Tumors measuring 2 to 4 cm remain a challenge. Latest data suggest that an abdominal radical trachelectomy should be performed.

## Data Availability

Not applicable.
